# Mitochondrial DNA Together with miR-142-3p in Plasma Can Predict Unfavorable Outcomes in Patients after Acute Myocardial Infarction

**DOI:** 10.3390/ijms23179947

**Published:** 2022-09-01

**Authors:** Teodora Barbalata, Alina I. Scarlatescu, Gabriela M. Sanda, Laura Toma, Camelia S. Stancu, Maria Dorobantu, Miruna M. Micheu, Anca V. Sima, Loredan S. Niculescu

**Affiliations:** 1 Lipidomics Department, Institute of Cellular Biology and Pathology “Nicolae Simionescu” of the Romanian Academy, 8, B.P. Hasdeu Street, 050568 Bucharest, Romania; 2 Department of Cardiology, “Carol Davila” University of Medicine and Pharmacy, 050474 Bucharest, Romania; 3 Department of Cardiology, Clinical Emergency Hospital of Bucharest, 014461 Bucharest, Romania

**Keywords:** biomarker, cardiovascular disease, cell-free DNA, microRNA, mitochondrial DNA, myocardial infarction

## Abstract

Myocardial infarction is one of the leading causes of death worldwide, despite numerous efforts to find efficient prognostic biomarkers and treatment targets. In the present study, we aimed to assess the potential of six microRNAs known to be involved in cardiovascular diseases, cell-free DNA (cfDNA), and mitochondrial DNA (mtDNA) circulating in plasma to be used as prognostic tools for the occurrence of unfavorable outcomes such as major adverse cardiovascular events (MACE) after acute ST-segment elevation myocardial infarction (STEMI). Fifty STEMI patients were enrolled and monitored for 6 months for the occurrence of MACE. Plasma was collected at three time points: upon admission to hospital (T_0_), at discharge from hospital (T_1_), and 6 months post-STEMI (T_6_). Plasma levels of miR-223-3p, miR-142-3p, miR-155-5p, miR-486-5p, miR-125a-5p, and miR-146a-5p, as well as of cfDNA and mtDNA, were measured by RT-qPCR. Results showed that the levels of all measured miRNAs, as well as of cfDNA and mtDNA, were the most increased at T_1_, compared to the other two time points. In the plasma of STEMI patients with MACE compared to those without MACE, we determined increased levels of miRNAs, cfDNA, and mtDNA at T_1._ Hence, we used the levels of all measured parameters at T_1_ for further statistical analysis. Statistical analysis demonstrated that all six miRNAs and cfDNA plus mtDNA levels, respectively, were associated with MACE. The minimal statistical model that could predict MACE in STEMI patients was the combination of mtDNA and miR-142-3p levels, as evidenced by ROC analysis (AUC = 0.97, *p* < 0.001). In conclusion, the increased plasma levels of mtDNA, along with miR-142-3p, could be used to predict unfavorable outcomes in STEMI patients.

## 1. Introduction

Cardiovascular diseases (CVD) are the main cause of death worldwide, despite countless efforts to find early and efficient prognostic and treatment methods. Among CVD events, acute ST-segment elevation myocardial infarction (STEMI) is one of the most dangerous ones. Because of its severity, recovery after STEMI is not always fully achieved, as some patients still remain at risk of developing future major adverse cardiovascular events (MACE), such as heart failure, myocardial infarction, or even death [1].

Current clinical parameters used are not accurate enough in predicting which patients are at risk of developing MACE after STEMI. There is therefore still a need for new early and efficient non-invasive markers that could complement classical parameters for this purpose. One promising class of molecules that could be used to predict the risk of developing MACE after STEMI is circulating cell-free DNA (cfDNA) and the mitochondrial fraction of cfDNA (mtDNA). MtDNA is well-known for its role in oxidative phosphorylation and inherited mitochondrial disorders [2]. Its immunoinflammatory role has only recently been recognized, mtDNA being proven as an activator for the innate immune system influencing the antimicrobial responses and inflammatory pathology. In the cytoplasm, extracellular space, or circulation, mtDNA can activate various pattern recognition receptors to trigger pro-inflammatory and type I interferon responses [3]. MtDNA that is not digested by autophagy could induce inflammation in the heart, a possible mechanism working in chronic non-infectious inflammation-related diseases such as atherosclerosis, metabolic syndrome, and diabetes mellitus [4]. It is known that both fractions of DNAs are markers of cardiac cell stress and death and their release in circulation is increased in pathological conditions [5,6]. However, only a few data exist regarding the prediction potential of cfDNA and mtDNA for post-STEMI events [7,8].

Another class of molecules, microRNAs (miRNAs), have been described as potential biomarkers for various diseases such as cancer, auto-immune diseases, and more recently CVD [9]. The use of miRNAs as prognostic markers for post-acute myocardial infarction (AMI) evolution is still under evaluation and clinical validation [10].

In the present study, we aimed to analyze the variation of six miRNAs (miR-223-3p, miR-142-3p, miR-155-5p, miR-486-5p, miR-125a-5p, miR-146a-5p) in the plasma of STEMI patients in correlation with their evolution at 6 months after STEMI. We selected this particular set of miRNAs based on the results of our previous studies on CVD patients [11,12,13,14]. We have previously shown that increased levels of miR-142-3p in the plasma and the atherosclerotic plaques of peripheral artery disease patients are associated with further cardiovascular events [12]. MiR-486-5p was demonstrated to be present in HDL particles and its HDL-associated levels were correlated with vulnerable coronary artery disease (CAD) [13]. We have also shown that miR-223-3p and miR-486-5p are the most abundant miRNAs in HDL_2_ [14]. All selected miRNAs were proven as good markers for CVD gravity, but their use in clinical practice as predictors for unfavorable outcomes is still at the beginning and should be further investigated.

The present study aimed to investigate the potential of circulating free DNA (cfDNA and mtDNA) and a set of miRNAs together with other clinical parameters to predict MACE in STEMI patients at 6-month follow-up. We prove for the first time that a combination between plasma mtDNA and miR-142-3p levels could significantly predict MACE in STEMI patients.

## 2. Results

### 2.1. Plasma Parameters of STEMI Patients; Variation at 6-Month Follow-Up

The plasma lipids parameters and glucose levels of STEMI patients at T_0_, T_1,_ and T_6_ time points and of the controls are given in Table S1, Supplementary Material. We determined a decreased highdensity lipoprotein-cholesterol/apolipoprotein A-I (HDL-C/apoA-I) ratio in the plasma of STEMI patients at T_0_ (by 23%, *p* = 0.007) and T_1_ (by 32%, *p* = 0.0004) and a significant increase at T_6_ (by 29%, *p* = 0.009) compared with the controls; this ratio was increased at T_6_ compared with T_0_ (by 60%, *p* = 3.60 × 10^−14^) and T_1_ (by 71%, *p* = 4.62 × 10^−14^), respectively (Figure S1a, Supplementary Material).

The atherogenic coefficient (nonHDL-C/HDL-C), an index that uses HDL and non-HDL cholesterol levels, was increased at T_0_ (by 87%, *p* = 0.04), T_1_ (by 67%, *p* = 0.003) compared with the controls and decreased at T_6_ compared with T_0_ (by 76%, *p* = 0.0006), and T_1_ (by 56%, *p* = 9.48 × 10^−6^), respectively (Figure S1b, Supplementary Material). If HDL-C/apoA-I ratio increased from levels below the controls (during hospitalization) to levels over this reference (at 6 months post-STEMI), mostly due to intense statin treatment, the atherogenic coefficient at 6 months returned to control levels.

The oxidative and inflammatory parameters in the plasma of STEMI patients at T_0_, T_1_, and T_6_ time points and of the controls are given in Table S2, Supplementary Material. Between the time points of post-STEMI follow-up, we found contradictory variations of oxidative status-related parameters. In the plasma of STEMI patients at T_1_ compared with T_0_, we measured significantly increased paraoxonase 1 (PON1) activity (1.9-fold, *p* = 5.55 × 10^−6^) and PON1 specific activity (1.6-fold, *p* = 6.44 × 10^−6^) (Table S2, Supplementary Material). However, myeloperoxidase (MPO) specific activity (1.74-fold, *p* = 0.0004) and ceruloplasmin (CP) (1.36-fold, *p* = 0.0002) levels increased at T_1_ compared with T_0_. Additionally, at T_6_ compared with T_0_ we detected increased PON1 activity (1.17-fold, *p* = 0.02) and PON1 specific activity (1.26-fold, *p* = 0.001), but also MPO specific activity (1.77-fold, *p* = 0.0007). In contrast, in plasma of STEMI patients at T_6_ compared with T_1_, decreased PON1 activity (1.61-fold, *p* = 0.002), MPO protein (1.36-fold, *p* = 0.0005), and CP (1.52-fold, *p* = 5.06 × 10^−6^) levels were measured (Table S2, Supplementary Material). At hospital discharge (T_1_), C-reactive protein (CRP) levels were increased (2.21-fold, *p* = 0.0003) compared with T_0_, whereas at T_6_ they were decreased compared with T_0_ (4.15-fold, *p* = 5.83 × 10^−8^) and T_1_ (9.2-fold, *p* = 4.49 × 10^−16^).

We further observed an increase in oxidative stress, expressed as MPO protein/PON1 protein ratio, as evidenced in the plasma of STEMI patients compared with the controls (at T_0_ by 3.15-fold, *p* = 0.0007, and at T_1_ by 2-fold, *p* = 0.021). This ratio was decreased at T_1_ (by 54%, *p* = 0.01) and T_6_ (by 70%, *p* = 0.0002) compared with T_0_ (Figure S2a, Supplementary Material).

The cardiac parameter measured in the plasma of STEMI patients and the controls was lactate dehydrogenase (LDH) (Table S2, Supplementary Material). Analysis of the time variation of LDH in the plasma of STEMI patients showed that at T_1_ compared with T_0_ there were lower levels (by 47%, *p* = 1.93 × 10^−5^). At 6 months after STEMI, LDH (2.53-fold, *p* = 9.79 × 10^−14^), levels were decreased compared with T_1_ (Table S2, Supplementary Material).

### 2.2. Plasma Levels of miRNAs in STEMI Patients; Variation at 6-Month Follow-Up

**Figure 1:**
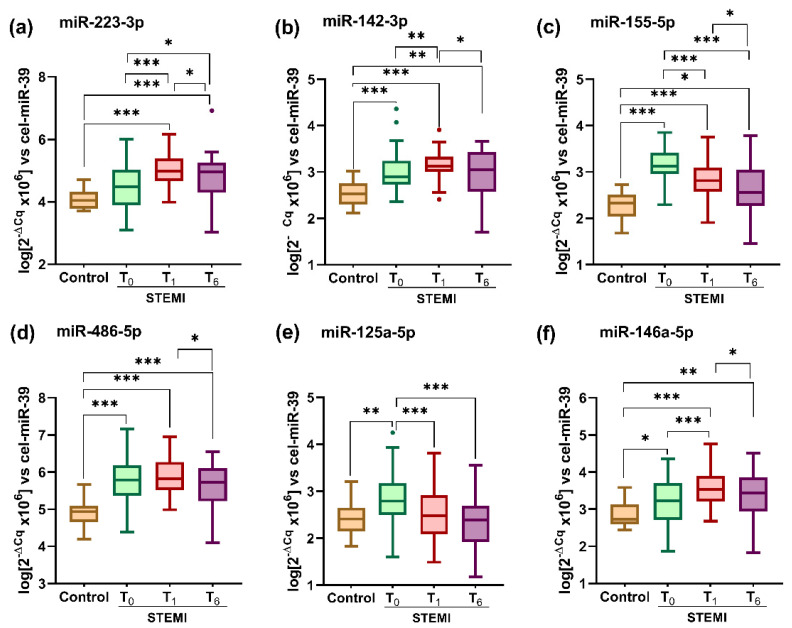
Levels of miRNAs (**a**–**f**) in the plasma of ST-segment elevation myocardial infarction (STEMI) patients at T_0_, T_1_, and T_6_ time points and of controls. Data are illustrated as boxplots with Tukey whiskers and median line. * *p* < 0.05; ** *p* < 0.01; *** *p* < 0.001.

We have measured the levels of six selected miRNAs known to be involved in CVD (miR-223-3p, miR-142-3p, miR-155-5p, miR-486-5p, miR-125a-5p, and miR-146a-5p) in plasma from STEMI patients at T_0_, T_1_, and T_6_ time points and from the controls (Figure 1a–f). The analysis of variation for the miRNA levels between the three time points of post-STEMI follow-up was conducted using the one-way ANOVA test. According to this analysis, significant variation trends during the post-STEMI follow-up were evidenced for plasma miR-223-3p levels (*p* = 6.18 × 10^−6^), miR-142-3p (*p* = 0.0275), miR-155-5p (*p* = 6.52 × 10^−10^), miR-125a-5p (*p* = 4.25 × 10^−7^), and miR-146a-5p (*p* = 1.41 × 10^−3^), but for miR-486-5p levels were not (*p* = 0.0561).

Compared with the controls, we found that all miRNA levels were increased in the plasma of STEMI patients at T_0_, T_1_, and T_6_ (Figure 1a–f). The only exception was miR-125a-5p, whose mean plasma level in STEMI patients at T_6_ returned to a mean level of controls, whereas the other five miRNAs remained still at higher levels.

Between the three time points of post-STEMI follow-up, we quantified different profiles for the distribution of measured plasma miRNA levels. At T_1_ compared with T_0_ the plasma levels of miR-223-3p (by 13%, *p* = 1.04 × 10^−6^), miR-142-3p (by 5%, *p* = 0.004) and miR-146a-5p (by 11%, *p* = 0.0003) were significantly increased, levels for miR-155-5p (by 10%, *p* = 9.11 × 10^−6^) and miR-125a-5p (by 14%, *p* = 0.0001) were significantly decreased, whereas miR-486-5p remained unchanged (Figure 1a–f). After 6 months post-STEMI (T_6_), we measured significantly decreased plasma levels for all miRNAs compared with T_0_ and T_1_: miR-223-3p (by 6% vs. T_0_, *p* = 0.01 and by 5% vs. T_1_, *p* = 0.03), miR-142-3p (by 5% vs. T_0_, *p* = 0.03), miR-155-5p (by 19% vs. T_0_, *p* = 5.27 × 10^−10^, and by 8% vs. T_1_, *p* = 0.01), miR-486-5p (by 4% vs. T_1_, *p* = 0.01), miR-125a-5p (by 22% vs. T_0_, *p* = 3.34 × 10^−7^) and miR-146a-5p (by 6% vs. T_1_, *p* = 0.04) (Figure 1a–f).

### 2.3. Plasma Levels of cfDNA and mtDNA in STEMI Patients; Variation at the 6-Month Follow-Up

**Figure 2:**
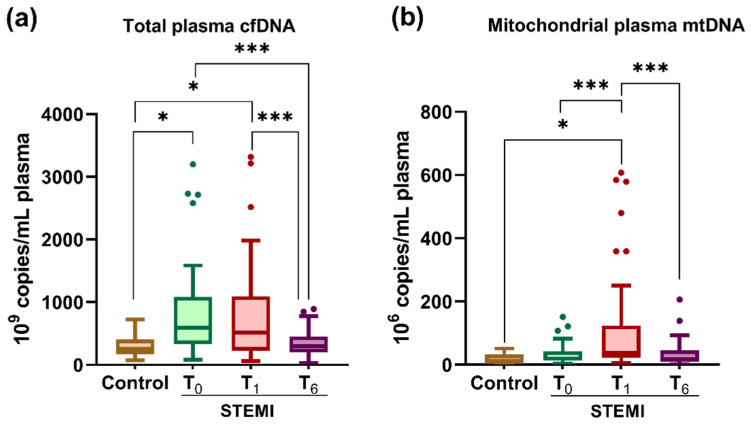
Levels of total cell-free (cfDNA) (**a**) and mitochondrial DNA (mtDNA) (**b**) in the plasma of ST-segment elevation myocardial infarction (STEMI) patients at T_0_, T_1_, and T_6_ time points and of healthy controls. Data are illustrated as boxplots with Tukey whiskers and median line. * *p* < 0.05; *** *p* < 0.001.

We analyzed the distribution of cfDNA and mtDNA in the plasma of STEMI patients at T_0_, T_1_, and T_6_ and of the controls (Figure 2). The analysis using a one-way ANOVA test showed that plasma cfDNA (*p* = 8.80 × 10^−5^) and mtDNA (*p* = 1.03 × 10^−6^) levels varied significantly between the three time points.

We determined that compared with the controls, plasma cfDNA levels were increased in STEMI patients at T_0_ (2.62-fold, *p* = 0.006) and T_1_ (2.75-fold, *p* = 0.01), and the levels of mtDNA were also increased at T_1_ (6-fold, *p* = 0.03) (Figure 2a,b). Among the three time points of the post-STEMI follow-up, results showed that the highest levels were recorded during hospitalization: cfDNA (both T_0_ and T_1_) and mtDNA (at T_1_). Then at T_6_, both cfDNA (by 2.23-fold vs. T_0_ *p* = 1.05 × 10^−5^ and by 2.34-fold vs. T_1_, *p* = 7.58 × 10^−5^) and mtDNA (3.45-fold vs. T_1_, *p* = 0.0007) levels were significantly decreased in the plasma of STEMI patients, returning to levels close to those of the controls (Figure 2a,b).

### 2.4. Correlations between Plasma Levels of miRNAs, cfDNA, mtDNA, and Main Plasma Parameters of STEMI Patients

**Figure 3:**
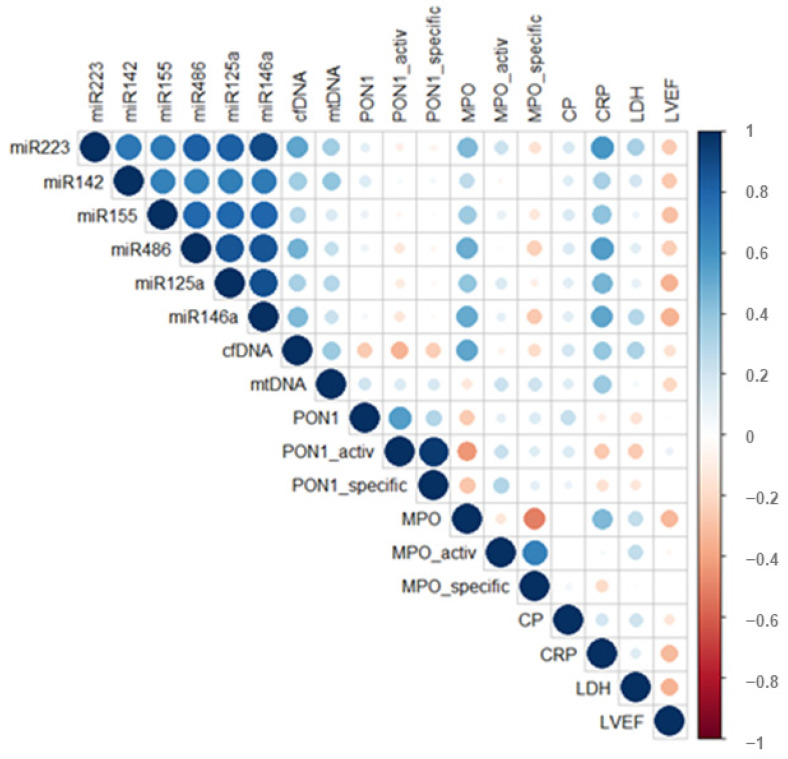
Correlation plots showing bivariate Spearman’s nonparametric correlations between the plasma miRNAs panel and cfDNA levels with patient’s oxidative, inflammatory, and cardiac parameters in ST-segment elevation myocardial infarction (STEMI) patients. Data are illustrated as correlation plots graphs using R-studio software.

Bivariate correlations of plasma miRNA, cfDNA, and mtDNA levels with oxidative and inflammatory stress and clinical parameters of STEMI patients at hospital discharge (T_1_) were estimated using Spearman’s nonparametric analysis and illustrated as correlation plots in Figure 3. Moderate positive correlations were observed for all six analyzed miRNA levels with cfDNA levels. Only three miRNAs positively and significantly correlated with mtDNA levels: miR-223-3p (R = 0.344, *p* = 0.017), miR-142-3p (R = 0.391, *p* = 0.005) and miR-125a-5p (R = 0.288, *p* = 0.042) (Figure 3).

After evaluating the potential associations of miRNA, cfDNA, and mtDNA levels with oxidative stress-related parameters in STEMI patients’ plasma, significant positive correlations were found between MPO protein levels and miR-146a-5p (R = 0.509, *p* < 0.001), miR-486-5p (R = 0.499, *p* < 0.001), miR-223-3p (R = 0.444, *p* = 0.002), miR-125a-5p (R = 0.390, *p* = 0.006), miR-155-5p (R = 0.353, *p* = 0.013), and cfDNA (R = 0.521, *p* < 0.001) levels (Figure 3).

When analyzing the associations of inflammatory stress parameters, we observed strong significant positive correlations between CRP levels with plasma levels of miR-223-3p (R = 0.587, *p* < 0.001), miR-486-5p (R = 0.557, *p* < 0.001), miR-146a-5p (R = 0.521, *p* < 0.001), miR-125a-5p (R = 0.465, *p* = 0.001), miR-155-5p (R = 0.404, *p* = 0.006), miR-142-3p (R = 0.327, *p* = 0.026), cfDNA (R = 0.382, *p* = 0.011), and mtDNA (R = 0.362, *p* = 0.017) in STEMI patients at T_1_. LDH levels correlated positively with miR-223-3p (R = 0.326, *p* = 0.027), miR-146a-5p (R = 0.282, *p* = 0.049), and cfDNA (R = 0.315, *p* = 0.029) (Figure 3). We further analyzed the potential associations of cardiac parameters with measured miRNA and cfDNA levels in the plasma of STEMI patients at T_1_, and we observed that the left ventricle ejection fraction (LVEF) showed significant negative correlations with miR-146a-5p (R = −0.350, *p* = 0.011), miR-125a-5p (R = 0.347, *p* = 0.014), and miR-155-5p (R = 0.295, *p* = 0.038) (Figure 3).

### 2.5. Clinical Parameters Associated with MACE at the 6-Month Follow-Up

**Figure 4:**
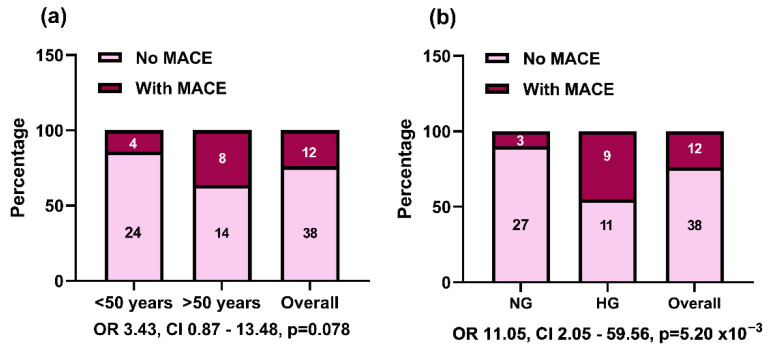
Distribution of major adverse cardiovascular events (MACE) occurrence at 6-month follow-up within age groups (below and over 50 years) (**a**) and hyperglycemic group (fasting glucose > 120 mg/dL during the hospital treatment) (**b**) in the ST-elevation myocardial infarction (STEMI) patients recruited for the study. The differences between group frequencies were analyzed by Pearson chi-square functions and Mantel–Haenszel common odds ratio (OR) estimates.

We analyzed the biochemical, clinical, epigenetic (miRNAs), cfDNA, and mtDNA parameters according to the occurrence of subsequent major adverse cardiovascular events (MACE) during the 6-month follow-up of STEMI patients. Additionally, the distribution of STEMI patients according to their age below or over 50 years (the median age) showed an increased incidence (by 80%, *p* = 0.070) of MACE in the group over 50 years, with a 3.4-fold higher risk of MACE occurrence compared with younger STEMI patients, but below statistical significance (Table 1 and Figure 4). Male patients were dominant in both follow-up groups being around 90% of STEMI patients with no statistical difference according to MACE occurrence (Table 1).

As expected, STEMI patients with MACE at the follow-up recorded lower ejection fractions at baseline compared with those without MACE (by 20%, *p* = 0.037, Table 1). About 3-fold more hyperglycemic STEMI patients developed MACE compared with those who did not have MACE (*p* < 0.001). Therefore, hyperglycemia was proven to be associated with an 11-fold higher risk (OR = 11.05, *p* = 5.2 × 10^−3^) for MACE occurrence compared with normoglycemic STEMI patients (Figure 4).

### 2.6. Plasma Parameters Associated with MACE at the 6-Month Follow-Up

The lipid metabolism-related parameters in plasma of STEMI patients at T_0_, T_1_, and T_6_ grouped by the occurrence of subsequent MACE during a 6-month follow-up are given in Table S3, Supplementary Material. When compared with STEMI patients without MACE, significantly decreased levels of HDL-C levels in STEMI patients with MACE (by 30% at T_0_, *p* = 0.004, and by 17% at T_1_, *p* = 0.002) were found. We determined significantly increased plasma levels of apoJ (by 9% at T_6_, *p* = 0.01) and Lp(a) (by 12% at T_1_, *p* = 0.03) in STEMI patients with MACE compared with those without MACE (Table S3, Supplementary Material).

The variation of the HDL-C/apoA-I ratio and atherogenic coefficient values in the plasma of STEMI patients at T_0_, T_1_, and T_6_ time points, grouped by the occurrence of subsequent MACE during a 6-month follow-up is shown in Figure S3, Supplementary Material. We observed significantly lower values of HDL-C/apoA-I ratio (by 68% at T_0_, *p* = 1.01 × 10^−7^) (Figure S3a, Supplementary Material), whereas no difference was recorded for the values of the atherogenic coefficient when comparing STEMI patients with MACE to those without MACE (Figure S3b, Supplementary Material).

The oxidative and inflammatory parameters in the plasma of STEMI patients at T_0_, T_1_, and T_6_ grouped by the occurrence of subsequent MACE during a 6-month follow-up are shown in Table S4, Supplementary Material. At T_1_, we observed augmented oxidative stress in STEMI patients with MACE compared with those without MACE, as revealed by the significantly decreased levels of PON1 protein (by 16%, *p* = 0.03), decreased PON1 activity (2-fold, *p* = 0.02) and significantly increased of MPO protein (by 43%, *p* = 0.04) (Table S4, Supplementary Material). We further observed that the MPO/PON1 protein ratio was significantly increased (by 1.8-fold at T_1_, *p* = 0.002). In contrast, no statistically significant changes were observed for the MPO/PON1 activity ratio (Figure S4a,b, Supplementary Material). Maximally augmented oxidative stress was evidenced at hospital discharge (T_1_) in STEMI patients with subsequent MACE. Plasma levels of the inflammatory stress parameter, CRP, were significantly increased (by 48% at T_0_, *p* = 0.04, 2-fold at T_1_, *p* = 9.22 × 10^−6^ and by 2-fold at T_6_, *p* = 0.007) (Table S4, Supplementary Material).

Significantly increased plasma LDH levels (by 1.51-fold at T_1_, *p* = 0.0005) were observed in STEMI patients with MACE compared with those without MACE (Table S4, Supplementary Material).

### 2.7. Association of Plasma miRNA Levels with MACE at the 6-Month Follow-Up

**Figure 5:**
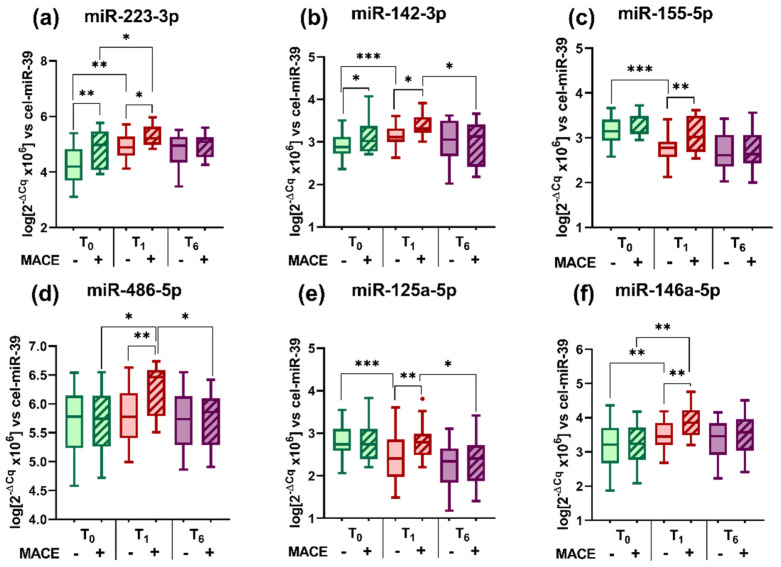
Variation of miRNA (**a**–**f**) levels in the plasma of ST-segment elevation myocardial infarction (STEMI) patients at T_0_, T_1_, and T_6_ time points grouped by the occurrence of subsequent major adverse cardiovascular events (MACE) at 6-month follow-up. Data are illustrated as boxplots with Tukey whiskers and median line; the “-” symbol signifies the respective STEMI group without MACE, while the “+” symbol signifies the respective STEMI group with MACE. * *p* < 0.05, ** *p* < 0.01, *** *p* < 0.001.

We analyzed the variation of the six selected miRNA levels in the plasma of STEMI patients at T_0_, T_1_, and T_6_ grouped by the occurrence of subsequent MACE during the 6-month follow-up (Figure 5). In STEMI patients with unfavorable outcomes (MACE) we measured significantly increased levels of: miR-223-3p (by 12% at T_0_, *p* = 0.006 and by 8% at T_1_, *p* = 0.041), miR-142-3p (by 6% at T_0_, *p* = 0.04 and by 6% at T_1_, *p* = 0.01), miR-155-5p (by 10% at T_1_, *p* = 0.006), miR-486-5p (by 7% at T_1_, *p* = 0.003), miR-125a-5p (by 17% at T_1_, *p* = 0.009), and miR-146a-5p (1.17-fold at T_1_, *p* = 0.006) (Figure 5a–f). No difference was measured for all miRNAs at 6 months post-STEMI in patients suffering MACE compared with those without MACE. At the 6-month follow-up (T_6_), these miRNAs returned or were even lower than the values recorded while in the hospital (T_0_ and T_1_) both in STEMI patients with and without MACE. Based on these data, we selected the values measured at the T_1_ time point for further statistical analysis.

### 2.8. Association of Plasma cfDNA and mtDNA Levels with MACE at the 6-Month Follow-Up

**Figure 6:**
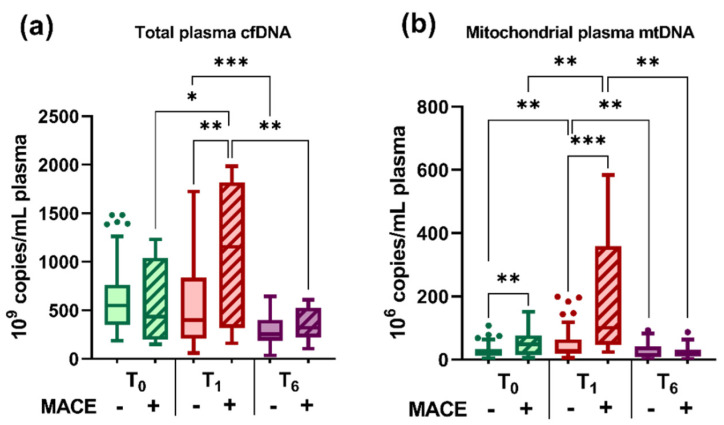
Variation of plasma levels of total cell-free (cfDNA) (**a**) and mitochondrial cfDNA (mtDNA) (**b**) in ST segment elevation myocardial infarction (STEMI) patients at T_0_, T_1_, and T_6_ time points grouped by the occurrence of subsequent major adverse cardiovascular events (MACE) at 6-month follow-up. Data are illustrated as boxplots with Tukey whiskers and median line; the “-” symbol signifies the respective STEMI group without MACE, while the “+” symbol signifies the respective STEMI group with MACE. * *p* < 0.05, ** *p* < 0.01, *** *p* < 0.001.

The variation of plasma levels of cfDNA and mtDNA in STEMI patients at T_0_, T_1_, and T_6_ grouped by the occurrence of subsequent MACE during the 6-month follow-up are illustrated in Figure 6. We observed significantly increased levels of plasma cfDNA (2-fold at T_1_, *p* = 0.005) and mtDNA (3.87-fold at T_1_, *p* = 0.0001) levels in STEMI patients with MACE as compared with those without MACE (Figure 6a,b).

### 2.9. Statistical Associations of Plasma Biochemical and Cardiac Parameters with Subsequent MACE in STEMI Patients

The binary logistic regression (BLR) analysis was used to estimate the risk of occurrence of subsequent MACE using lipid metabolism, oxidative, and inflammatory stress parameters measured in the plasma of STEMI patients at hospital discharge (T_1_) as potential predictors; all BLR models were adjusted for age and gender (Table S5, Supplementary Material). The reference category was considered as in the “no MACE” group. Using some lipid metabolism-related parameters, the age and gender-adjusted BLR model 1 could significantly predict the MACE during the 6-month follow-up of STEMI patients (χ^2^ = 17.52, *p* < 0.001) (BLR model 1, Table S5, Supplementary Material). The important contributions to this adjusted BLR model 1 were given by the HDL-C/apoA-I ratio (Wald χ^2^ = 3.68, *p* = 0.055) and the atherogenic coefficient (Wald χ^2^ = 3.47, *p* = 0.062).

Another BLR model using some oxidative stress-associated parameters (PON1 and MPO protein and activity levels) could significantly predict the subsequent MACE in STEMI patients (χ^2^ = 22.58 for model 2, *p* < 0.001), but the model may be affected after the adjustment made for gender (highest Wald χ^2^ = 3.68 in the model) in this prediction (BLR model 2, Table S5, Supplementary Material). A strong MACE prediction could be conducted using a BLR model involving some inflammatory stress-related parameters in STEMI patients (χ^2^ = 32.96 for model 3, *p* < 0.001), where CRP and LDH have high individual scores (Wald χ^2^ = 3.97, *p* = 0.046, and Wald χ^2^ = 3.54, *p* = 0.060), whereas the adjustment for age (Wald χ^2^ = 5.33, *p* = 0.021) could influence this model (BLR model 3, Table S5, Supplementary Material). The clinical data and risk factors of STEMI patients could significantly predict the subsequent MACE during their follow-up (χ^2^ = 52.32 for model 4, *p* < 0.001), the most powerful contributors to the model being hyperglycemia (Wald χ^2^ = 5.34, *p* = 0.021), LVEF (Wald χ^2^ = 4.35, *p* = 0.037), and dyslipidemia (Wald χ^2^ = 1.62, *p* = 0.202), with a moderate influence after adjustment for gender (Wald χ^2^ = 2.14, *p* = 0.143) (BLR model 4, Table S5, Supplementary Material).

**Figure 7:**
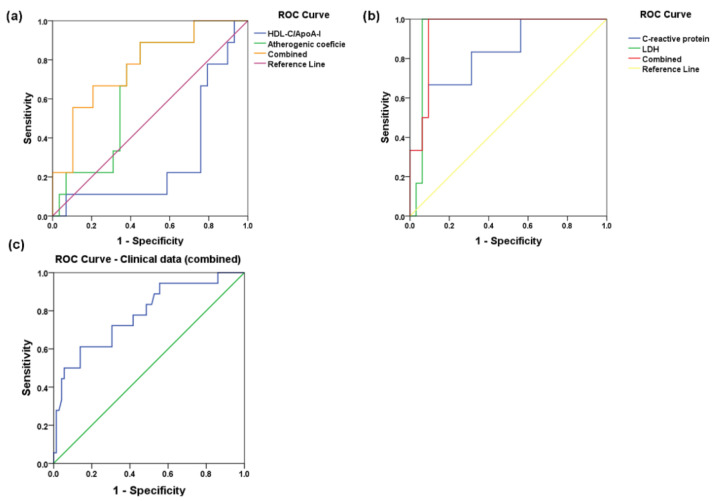
Receiver operating characteristic (ROC) curves for the predictive potential of univariate and multivariate models for follow-up major adverse cardiovascular events (MACE) using: (**a**) HDL-C/apoA-I ratio and atherogenic coefficient (non-HDL-C/HDL-C), (**b**) C-reactive protein (CRP) and lactate-dehydrogenase (LDH) levels, and (**c**) clinical data, plasma values being measured at hospital discharge (T_1_) in plasma from ST-elevation myocardial infarction (STEMI) patients. All diagonal lines are for AUC = 0.5.

We used the receiver operating characteristic (ROC) analysis, adjusted for age and gender, to estimate the prediction potential of each of the biochemical and clinical parameters for the subsequent MACE occurrence during the 6-month follow-up of STEMI patients. A multivariate model using two lipid metabolism parameters, HDL/apoA-I ratio, and the atherogenic coefficient proved to be powerful independent predictors of MACE incidence in STEMI patients [area under the curve (AUC) = 0.77, *p* = 0.015] in multivariate ROC, adjusted for age and gender (multivariate model 1 in Table 2 and Figure 7a). In addition, the univariate ROC analysis showed that both the HDL-C/apoA-I ratio and atherogenic coefficient individually failed to reach statistical significance (Table 2 and Figure 7a).

The inflammatory stress-related parameters CRP and LDH proved to be significant predictors, both individually in univariate ROC analysis (AUC = 0.86 and AUC = 0.94, both *p* < 0.001) and when combined in multivariate ROC analysis (AUC = 0.95, *p* < 0.001) (multivariate model 2 in Table 2 and Figure 7b). The combination model using classical risk factors (dyslipidemia, hyperglycemia, hypertension, obesity, smoking) and clinical data (LVEF) can significantly predict subsequent MACE in STEMI patients in a multivariate ROC analysis adjusted for age and gender (AUC = 0.78, *p* < 0.001) (multivariate model 3 in Table 2 and Figure 7c), but with lower confidence compared with the multivariate model using inflammatory stress-related parameters.

### 2.10. Plasma-Selected miRNA, cfDNA, and mtDNA Levels as Predictors for Subsequent MACE in STEMI Patients

We further used BLR analysis to estimate the occurrence of subsequent MACE using the miRNA, cfDNA, and mtDNA levels measured in the plasma of STEMI patients at hospital discharge (T_1_) as potential predictors. Using the plasma levels of all six analyzed miRNAs, the unadjusted BLR model could significantly predict the MACE during the 6-month follow-up of STEMI patients (χ^2^ = 20.82, *p* < 0.001) (BLR model 1, Table S6, Supplementary Material). The high-ranking contributions to this adjusted BLR model 1 were added by miR-125a-5p (Wald χ^2^ = 3.79, *p* = 0.052), miR-486-5p (Wald χ^2^ = 3.61, *p* = 0.057), and miR-146a-5p levels (Wald χ^2^ = 2.21, *p* = 0.137) (Table S6, Supplementary Material).

The addition of the two novel circulating markers in BLR model 2, cfDNA and mtDNA levels, increased the prediction potential for follow-up MACE occurrence in STEMI patients (χ^2^ = 40.57, *p* < 0.001) (BLR model 2, Table S6, Supplementary Material). The highest-ranking contributor in this BLR model was plasma mtDNA (Wald χ^2^ = 3.86, *p* = 0.049), together with cfDNA and levels of the three high-ranked miRNAs from model 1 (miR-486-5p, miR-125a-5p and miR-146a-5p). The adjustment made for age and gender for BLR analysis did not significantly affect the strong forecast of the follow-up MACE incidence in STEMI patients using the plasma levels of all six analyzed miRNAs, cfDNA, and mtDNA (χ^2^ = 41.74, *p* < 0.001) (BLR model 3, Table S6, Supplementary Material).

**Figure 8:**
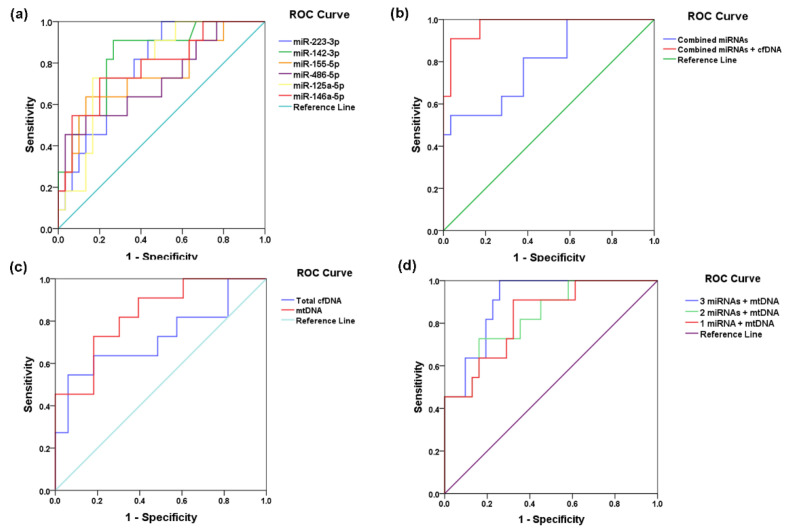
Receiver operating characteristic (ROC) curves for predictive potentials of individual and combined models for major adverse cardiovascular events (MACE) using: (**a**) individual miRNAs, (**b**) multivariate models of all 6 miRNAs and with cell-free DNA (cfDNA and mtDNA) levels, (**c**) total cell-free DNA (cfDNA), and circulating mitochondrial DNA (mtDNA), and (**d**) minimal combined models of one, two and three miRNAs and mtDNA levels; the values were measured at hospital discharge (T_1_) in plasma of ST-elevation myocardial infarction (STEMI) patients.

We applied the univariate ROC analysis, adjusted for age and gender, to estimate the prediction potential of each of the six miRNAs related to lipid metabolism, cfDNA, and mtDNA levels measured at T_1_ for the follow-up MACE occurrence in STEMI patients. Each of the six analyzed miRNAs could be used as individual predictors of subsequent MACE in STEMI patients in univariate ROC analysis, whereas the miR-142-3p was proven to be the strongest independent predictor (highest AUC = 0.832, *p* < 0.001), followed by miR-223-3p (AUC = 0.788, *p* < 0.001), and miR-146a-5p (AUC = 0.785, *p* < 0.001) (Table 3 and Figure 8a). Additionally, the multivariate ROC analysis indicated that the combination of the six selected miRNAs could strongly predict unfavorable outcomes in STEMI patients (AUC = 0.796, *p* < 0.001) (multivariate model 1 in Table 3 and Figure 8b).

Univariate ROC analysis showed that plasma mtDNA levels measured at T_1_ are stronger predictors of MACE in STEMI patients (AUC = 0.832, *p* < 0.001) than cfDNA levels (AUC = 0.722, *p* = 0.029) (Table 3 and Figure 8c). The addition of cfDNA and mtDNA levels to the combination model using the six miRNA levels significantly improved the multivariate ROC prediction (AUC = 0.97, *p* < 0.001) (multivariate model 2 in Table 3 and Figure 8c). We designed a minimal prognostic model using miRNA and mtDNA plasma levels as parameters measured at hospital discharge (T_1_) that can significantly predict MACE occurrence. We found that a combination of plasma levels of mtDNA and miR-142-3p is the best minimal significant multivariate ROC model (AUC = 0.97, *p* < 0.001) that can still predict unfavorable outcomes (multivariate model 3 in Table 3 and Figure 8d). Adding one (mi-223-3p) or two (mi-223-3p and miR-146a-5p) miRNAs, from those having high univariate AUCs, to this minimal ROC model, does not modify the MACE prediction potential, whereas it slightly increased for the model with mtDNA and three miRNAs (ROC model 4 and model 5 in Table 3 and Figure 8d).

## 3. Discussion

In this study, we assessed the potential of new markers such as extracellular non-coding RNA (miRNAs) and circulating cfDNA and mtDNA in association with oxidative and inflammatory stress parameters to predict unfavorable outcomes in STEMI patients. We measured their circulating levels at different time points and correlated these data with MACE occurrence during the 6-month follow-up. As far as we know, this is the first longitudinal prospective study to assess the prognostic potential of circulating cfDNA, mtDNA, and of CVD-related miRNAs, along with plasma biochemical parameters to predict the subsequent MACE in STEMI patients.

Our data show that STEMI patients at their hospital discharge (T_1_) compared with hospital admission (T_0_) have: (i) an improved oxidative status as the pro-oxidant MPO/antioxidant PON1 protein ratio is decreased and the PON1 activity is increased; (ii) an altered inflammatory status as the CRP level is increased; (iii) an altered cardiovascular status with an increased plasma LDH; (iv) increased plasma levels for all six miRNAs analyzed (miR-223-3p, miR-142-3p, miR-155-5p, miR-486-5p, miR-125a-5p, and miR-146a-5p); (v) and increased plasma levels of cfDNA and mtDNA.

All these parameters were evaluated for their association with the occurrence of MACE during the follow-up. Therefore, we assessed the potential of epigenetic (miRNA) markers and mtDNA measured at the hospital discharge (T_1_) to predict the occurrence of MACE. Compared with STEMI patients without MACE, those with MACE had: (i) augmented oxidative stress as revealed by the decreased levels of PON1 protein, PON1 activity, increased MPO protein, and MPO/PON1 protein ratio; (ii) augmented inflammatory stress as CRP levels increased; (iii) an increased plasma LDH associated with altered cardiovascular status; (iv) increased levels of all selected miRNAs (miR-223-3p, miR-142-3p, miR-155-5p, miR-486-5p, miR-125a-5p, and miR-146a-5p); (v) and increased levels of cfDNA and mtDNA. According to the statistical BLR and ROC analysis, we report here for the first time that the plasma levels of mtDNA together with miR-142-3p at T_1_ can predict with very good accuracy unfavorable outcomes in STEMI patients.

The set of six miRNAs (miR-223-3p, miR-142-3p, miR-155-5p, miR-486-5p, miR-125a-5p, miR-146a-5p) was selected based on the results of our previous studies regarding CVD patients [11,12,13,14] and an atherosclerosis animal model [15]. The predictive potential of these miRNAs using ROC curve analysis shows the highest univariate AUC calculated for miR-142-3p, miR-223-3p, and miR-146a-5p. Additionally, the minimal prediction model based on ROC analysis is obtained for mtDNA together with miR-142-3p. Although miR-223-3p and miR-146-4p proved to have an important univariate contribution to MACE prediction, they did not improve the prediction potential of the minimal model composed of mtDNA and miR-142-3p.

The powerful discrimination ability of plasma miR-142-3p as an independent predictor for subsequent MACE in the studied STEMI patients is one important result of the present study. Our data are in good agreement with Tang et al. who reported that increased levels of plasma miR-142-3p are associated with myocardial infarction in dual antiplatelet-treated patients undergoing percutaneous coronary intervention [16]. The study of Pan et al. reveals that circulating miR-142-5p levels may predict the development of in-stent restenosis in stent-implanted patients [17]. In addition, miR-142-3p is proposed as a key regulator of cardiomyocyte fibrosis and apoptosis induced by hypoxia/reoxygenation, thus serving as a candidate for therapeutic strategy in the management of patients suffering from myocardial infarction [18]. We recently showed that increased levels of miR-142-3p in the femoral atheroma from peripheral artery disease patients are associated with the appearance of further cardiovascular events [12]. This increased plaque expression of miR-142-3p positively correlated with plaque pri-miR-142 expression and plasma miR-142-3p levels, suggesting a local vascular production of miR-142 in the atheroma and its possible secretion in the blood. Functional bioinformatic analysis using the Database for Annotation, Visualization and Integrated Discovery (DAVID) platform shows that the potential gene targets for miR-142-3p are associated with 46 various biological processes including the Wnt signaling pathway, calcium modulating pathway, regulation of cytokine production involved in the inflammatory response, insulin receptor signaling pathway, and regulation of protein phosphorylation (for detailed gene ontology biological processes see [12]).

Circulating cell-free DNA (cfDNA) has recently evolved as a promising biomarker in acute CVD and as a mortality predictor in myocardial infarction. Accordingly, cfDNA is proposed as an additional early biomarker for cardiometabolic risk assessment and for arterial stiffness [19]. The prospective study of Gornik et al. shows that cfDNA levels measured at intensive care unit admission and 24 h after admission for non-traumatic cardiac arrest are associated with intra-hospital mortality [20]. However, these prospective studies conclude that longitudinal studies are needed to ascertain whether the baseline (intra-hospital) cfDNA levels are predictive for future MACE. There are only a few studies analyzing this aspect. A study associating circulating cfDNA with clinical manifestations demonstrates that the short-term (1–5 days) monitoring of AMI (STEMI and NSTEMI) patients correlates with the disease severity [7]. The same study shows that medium-term (1–5 months) monitoring indicates the existence of two distinctive groups, one having cfDNA returned to basal levels and the other with persistently elevated cfDNA levels. The novelty of our study is that cfDNA levels measured in the plasma of STEMI patients are increased during the short-term hospitalization (T_1_ vs. T_0_) and return to basal levels at a 6-month follow-up, and the values from hospital discharge (T_1_) can independently predict the occurrence of MACE during the 6-month follow-up. In good agreement with our data, the study of Borissoff et al. reports elevated levels of extracellular cfDNA, which are independently associated with the severity, extent, and phenotype of coronary atherosclerosis and with MACE incidence [5].

The levels of peripheral blood leukocyte mtDNA are proposed as a biomarker for evaluating and predicting CAD severity in several studies, providing improved patient discrimination [6,21]. A recent study demonstrates that the mtDNA copy number in the blood may serve as a biomarker for CVD-related phenotypes [22]. However, few studies have investigated the use of mtDNA levels (or copy number) as a prognostic marker for ischemic heart disease evolution. The most valuable and novel result of our study is the demonstration in a longitudinal prospective study that mtDNA levels measured at hospital discharge (T_1_) are independently associated with MACE occurrence during a 6-month follow-up post-STEMI that may have potential clinical utility in improving MACE risk prediction. Moreover, the maximal levels of mtDNA measured in STEMI patients having subsequent MACE are reached at the hospital discharge (T_1_). In good agreement with our results, Cosentino et al. suggest that cytochrome c and mtDNA may be associated with acute ventricular dysfunction and with short-term and long-term outcomes [23]. Another observational trial shows that the increase in free circulating mtDNA in the blood is a predictor of lethal outcomes in patients with acute coronary syndrome [24]. Interestingly, our data show good correlations of mtDNA levels with plasma levels and miR-142-3p in STEMI patients.

Herbers et al. show that the mitochondrial transcripts are most abundant in tissues with a high metabolic rate, but the mtDNA copy number per tissue mass is similar in all tissues [25]. The tissue-specific mtDNA content is controlled by the intrinsic reactive oxygen species exposure and mediated by DNA repair factors. However, less is known about the adverse effects on cell function that could be induced by extracellular mtDNA. Bliksøen et al. demonstrate that extracellular mtDNA isolated from AMI patients’ sera can be internalized, activating NF-kB via toll-like receptor 9 (TLR9) causing mitochondrial dysfunction and cardiomyocyte death [26]. They also suggest that endogenous mtDNA presence/secretion in the extracellular space is a danger signal with direct detrimental effects on cardiac cells. The in vivo study of Oka et al. demonstrates that the inhibition of mtDNA degradation by autophagy leads to inflammation and cardiac dysfunction through TLR9-mediated mechanisms in pressure-overloaded heart failure in mice [4]. It was shown that cellular mtDNA alterations lead to mitochondrial dysfunction, promoting inflammatory stress and cell senescence, these processes being considered proatherogenic [27]. Accordingly, mtDNA damage may be associated with atherosclerosis, and it is thought to play an important role in the initiation and development of CVD. It was shown that focal myocardial necrosis due to AMI induces the mtDNA release and that mtDNA plasma levels positively correlate with the extent of myocardial damage [28].

One potential limitation of our study is the relatively small number of STEMI patients analyzed. Although the number of recruited STEMI patients was rather small, the employed statistical analyses and BLR or ROC models, as well as the variation of the studied miRNAs set and of cfDNA/mtDNA levels during the follow-up of STEMI patients, revealed statistically significant data. We also explored the contribution of some important risk factors (age, gender, hyperglycemia, hypertension, dyslipidemia, obesity, and smoking), considering them to be cofactors in the statistical models. A clearly positive aspect is the accurate PCR method used for the quantification of cfDNA/mtDNA levels on a specific standard curve and data expressed as copies/mL plasma.

## 4. Materials and Methods

### 4.1. Study Design: STEMI Patients and Control Group

Our longitudinal prospective study included 50 STEMI patients who were treated by primary percutaneous coronary intervention (PCI), enrolled at the Emergency Hospital “Floreasca”, Bucharest, Romania. Patients with previous myocardial infarction, cardiac surgery, active malignancy, autoimmune diseases, severe hepatic/respiratory/renal failure, recent surgery or trauma, patients with addictions, poor compliance, or those who refused/or were unable to sign the informed consent were excluded. We did not include ICU patients with cardiogenic shock or cardiac arrest in our study.

The patients were monitored for 6 months for occurrence of post-STEMI unfavorable outcomes (MACE). The primary endpoint of our study was MACE defined according to the published European Society of Cardiology (ESC) Clinical Practice Guidelines [29] and recent literature [1] as recurrent ischemia/reinfarction requiring hospital admission and repeated PCI; heart failure requiring hospital readmission or outpatient treatment adjustment; death from cardiovascular causes. Reinfarction was defined according to the fourth universal definition of infarction guidelines and STEMI guidelines [30,31] as positive cardiac enzymes associated with at least one of the following (ischemia symptoms, new electrocardiogram changes suggestive of ischemia, new wall motion abnormality at cardiac imaging consistent with ischemic etiology). Heart failure was defined as symptoms suggestive of heart failure combined with LVEF dysfunction (EF < 49%) according to ESC guidelines [32]. Angina (chest pain) alone was not considered an endpoint. Because of the COVID-19 pandemic, the study group was reduced to 50 STEMI patients who responded to our call for follow-up: aged between 30 and 75 years old, including 22 males and 28 females. The patients were divided in 2 groups: “no MACE” (*n* = 38) and “with MACE” (*n* = 12).

Blood was collected on EDTA-K2 tubes from all patients at 3 time points: at admission to hospital (T_0_), at discharge from hospital (T_1_), and 6 months post-STEMI (T_6_); all the samples were processed to isolate plasma by centrifugation at 2000× *g*, 10′, at 4 °C. The plasma samples were aliquoted and stored at −80 °C until further processing. A control group was constituted comprising 14 healthy volunteers, free of cardiovascular disease, from the Institute of Cellular Biology and Pathology “Nicolae Simionescu” in Bucharest, Romania. Their fasting plasma was collected and stored at −80 °C until further processing.

The study was carried out adhering to the principles of the Declaration of Helsinki (The Code of Ethics of the World Medical Association, last updated at the 64th WMA General Assembly, Fortaleza, Brazil, October 2013) for experiments involving humans. All participants gave their written informed consent by signing the appropriate paperwork and respecting their anonymity and privacy rights; they were allocated a number in the study database. The Ethics Committees of the Institute of Cellular Biology and Pathology “Nicolae Simionescu” (#03/8 October 2019) and of the Clinical Emergency Hospital of Bucharest (#8575/24 September 2019) approved the study.

### 4.2. Determination of Plasma Parameters of STEMI Patients and Controls

The plasma parameters of STEMI patients and controls were measured using commercial kits: total cholesterol, HDL-C, low-density lipoprotein-cholesterol (LDL-C), triglycerides, and glucose from Dialab, Neudorf, Austria; apoA-I and apoE from Mabtech, Nacka Strand, Sweden; ELISA kits for apoJ (known as clusterin), PON1, MPO, and CRP from R&D Systems, Minneapolis, MN, USA; ELISA kit for Lp(a) from Novus Biologicals, Littleton, CO, USA; MPO enzymatic activity kit from Cayman Chemical, Ann Arbor, MI, USA; ELISA kit for CP from Abnova, Taipei, Taiwan; enzymatic activity of LDH from Biovision, Waltham, MA, USA. All assays were performed according to the manufacturer’s instructions. PON1 enzymatic activity was measured using an adapted method in the plasma of the patients [33]. Glucose levels at T_0_ and T_1_ were measured by the hospital’s laboratory. PON1- and MPO-specific activities were calculated as ratios between enzymatic activity and corresponding protein levels.

### 4.3. Analysis of microRNAs in the Plasma of STEMI Patients and Controls

Plasma miRNAs were isolated using miRNeasy Serum/Plasma kit (Qiagen, Hilden, Germany) following the manufacturer’s instructions. Twenty-five fmoles of synthetic cel-miR-39 (Life Technologies, Carlsbad, CA, USA) were added to each sample as a spike-in to correct the sample-to-sample variation, as we previously described [11,12].

There is an inherent association between plasma miRNA and cfDNA/mtDNA levels and heparinization of plasma (heparinization is required for PCI in STEMI patients). Before reverse transcription reaction, all isolated miRNA samples from T_0_ were treated with heparinase (Applied Biosystems, Waltham, MA, USA) to eliminate any residual heparin or fractioned heparin after the standard treatment of STEMI patients. The plasma expressions of hsa-miR-223-3p (ID 002295), hsa-miR-142-3p (ID 000464), hsa-miR-155-5p (ID 002623), hsa-miR-486-5p (ID 001278), hsa-miR-125a-5p (ID 002198), and hsa-miR-146a-5p (ID 000464) were measured using TaqMan miRNA Assays (Thermo Scientific, Waltham, MA, USA) on ViiA7 Real-Time PCR System (Applied Biosystems, USA) as we previously reported [11,12]. The expression level of each individual miRNA was determined relative to that of exogenously added cel-miR-39 (ID 000200) and calculated by using the 2^−ΔDCq^ method [12].

### 4.4. Analysis of cfDNA and mtDNA in the Plasma of STEMI Patients and Controls

Plasma DNA was isolated using QIAamp DNA Blood Mini kit (Qiagen), following the manufacturer’s instructions. All isolated DNA samples from T_0_ were treated with heparinase (Applied Biosystems, USA) to eliminate any residual heparin or fractioned heparin after the standard treatment of STEMI patients. CfDNA was measured as expression of LINE-1 (Long Interspersed Element class 1, which is a transposable element) by TaqMan technology (Thermo Scientific) using an adapted method described by Yu et al. [34], whereas mtDNA was measured as expression of MT-ND1 (mitochondrial NADH Dehydrogenase subunit 1) by SYBR Green-based qPCR (Applied Biosystems) using a modified method described by Lindqvist et al. [35]. The primer sequences used are given in Table S7, Supplementary Material. The data calculated for cfDNA and mtDNA were expressed as number of copies of DNA/mL of plasma using a standard curve (0.1–10,000 pg/well for LINE1 and 0.001–100 pg/well for MT-ND1) generated from specific PCR products using total DNA isolated from peripheral blood mononucleated cells from a healthy volunteer. For the conversion from pg DNA/mL to copies DNA/mL we used an online tool.

### 4.5. Statistical Analysis

Statistical analysis and graphical representations were conducted using the statistical software SPSS for Windows v21.0 (IBM SPSS, IBM Ireland, Dublin, Ireland) and GraphPad Prism 9.0 (GraphPad Software Inc., San Diego, CA, USA). Correlation plots were designed with Statistical Software Package R 4.0.3 (particularly *xlsx* and *corrplot* packages) and R-studio for Windows (version 1.3.1093). The continuous distributed quantitative variables (biochemical, miRNA, cfDNA, and mtDNA data) were expressed as means ± standard error of the mean (SEM) and analyzed by independent Student’s *t*-test for the comparison between study groups. Crosstabs distribution with chi-squared (χ^2^) analysis with Mantel–Haenszel common odds ratio (OR) estimates was performed by SPSS to evaluate the differences between categorical variables (gender, age distribution, presence of hyperglycemia, hypertension, dyslipidemia, obesity, and smoking). For statistical analysis, the values of plasma miRNA levels were log-transformed. Spearman’s nonparametric bivariate correlation analysis was performed using SPSS for plasma miRNA, cfDNA, and mtDNA levels with biochemical parameters. To analyze the potential of plasma miRNAs, cfDNA, and mtDNA to predict subsequent MACE, we employed a binary logistic regression (BLR) model with the enter iteration method, considering “no MACE” group as reference category, and “with MACE” group as risk (vulnerable) category, adjusting for age and gender (with females as reference group). ROC curve analysis (2-value) was used to estimate the prediction ability of a subsequent MACE for plasma miRNA, cfDNA, and mtDNA levels; univariate and multivariate ROC curve analysis was used for all six analyzed miRNAs, cfDNA, and mtDNA, adjusted for age and gender. The threshold for statistical significance was set to 5% (*p*-values lower than 0.05).

## 5. Conclusions

Our data based on high circulating extracellular miR-142-3p and mtDNA levels measured at hospital discharge support the clinical use of a valid and practical model to assess the risk of unfavorable outcomes (MACE) within the first 6 months after STEMI. These parameters are easy to quantify in plasma by using a fast and reliable molecular biology method (TaqMan-based PCR), available in most clinical hospitals. The traditional prediction based on other biochemical parameters (CRP and LDH) and clinical data (“classical” risk factors) remain important, but the risk prediction using new epigenetic (miR-142-3p) and mtDNA (as a marker of cardiac injury) is more confident and precise.

## Figures and Tables

**Table 1:** Distribution of clinical data, CVD risk factors, and angiographic characteristics of ST-segment elevation myocardial infarction (STEMI) patients overall and grouped by the occurrence of subsequent major adverse cardiovascular events (MACE) at 6-month follow-up.

Parameter	All STEMI with Follow-Up (*n* = 50)	No MACE (*n* = 38)	With MACE (*n* = 12)	*p*-Value
**Clinical characteristics**				
Age (years)	49.36 ± 1.27	49.34 ± 1.61	49.42 ± 1.49	0.641
Aged group (>50 years old), *n* (%)	22 (44.0)	14 (36.8)	8 (66.7)	0.070
Gender (male), *n* (%)	45 (90.0)	34 (89.5)	11 (91.7)	0.825
LVEF (%)	37.96 ± 1.32	39.38 ± 1.27	32.70 ± 3.78	0.037
**Cardiovascular risk factors**				
Hyperglycemia, *n* (%)	20 (40.0)	11 (28.9)	9 (75.0)	1.68 × 10^−3^
Hypertension, *n* (%)	28 (56.0)	19 (50.0)	9 (75.0)	0.128
Dyslipidemia, *n* (%)	44 (88.0)	32 (84.2)	12 (100.0)	0.142
Obesity, *n* (%)	12 (24.0)	10 (26.3)	2 (16.7)	0.495
Smoking, *n* (%)	45 (90.0)	35 (92.1)	10 (83.3)	0.377
**Angiographic characteristics**				
LAD, *n* (%)	21 (42.0)	17 (44.7)	4 (33.3)	0.341
RCA, *n* (%)	20 (40.0)	13 (34.2)	7 (58.3)	
LCX, *n* (%)	9 (18.0)	8 (21.1)	1 (8.3)	

Data are given as mean ± SEM (for age) and as case numbers (and percent) for clinical features. Statistical difference between groups was assessed either by independent Student’s t-test, Mann–Whitney U test (for continuous data age, LVEF), or Pearson χ^2^ analysis for categorical data (clinical and angiographic data, cardiovascular risk factors). LVEF, left ventricle ejection fraction; LAD, left anterior descending artery; RCA, right coronary artery; LCX, left circumflex artery.

**Table 2:** Receiver operating characteristic (ROC) analysis for the predictive potential of univariate and multivariate (combined) models for subsequent major adverse cardiovascular events (MACE), using clinical data, HDL-C/apoA-I ratio, atherogenic coefficient (non-HDL-C/HDL-C), C-reactive protein (CRP) and lactate-dehydrogenase (LDH) values measured at hospital discharge (T_1_) in plasma from ST-elevation myocardial infarction (STEMI) patients.

Area under the Curve					
Test Result Variable(s) *	Area	Standard Error ^a^	*p*-Value ^b^	Asymptotic 95% Confidence Interval	
				Lower Bound	Upper Bound
**Univariate models**					
HDL-C/apoA-I ratio	0.299	0.101	0.071	0.102	0.496
Atherogenic coefficient (AC)	0.667	0.092	0.135	0.486	0.848
CRP	0.860	0.076	1.77 × 10^−3^	0.711	1.000
LDH	0.943	0.040	6.67 × 10^−4^	0.864	1.000
**Multivariate model 1** (HDL/ApoA-I ratio and AC)	0.770	0.089	0.015	0.595	0.945
**Multivariate model 2** (CRP and LDH)	0.953	0.038	6.67 × 10^−4^	0.869	1.000
**Multivariate model 3** (dyslipidemia, hyperglycemia, hypertension, obesity, smoking and LVEF)	0.780	0.065	2.46 × 10^−4^	0.653	0.908

* All adjusted for age and gender (male). ^a^ Under the nonparametric assumption. ^b^ Null hypothesis: true area = 0.5.

**Table 3:** Receiver operating characteristic (ROC) analysis for predictive potentials of univariate and multivariate models for follow-up major adverse cardiovascular events (MACE), using plasma miRNA, total cell-free DNA (cfDNA), and cell-free mitochondrial DNA (mtDNA) values measured at hospital discharge (T_1_) in ST-elevation myocardial infarction (STEMI) patients.

Area under the Curve					
Test Result Variable(s) *	Area	Standard Error ^a^	*p*-Value ^b^	Asymptotic 95% Confidence Interval	
				Lower Bound	Upper Bound
**Univariate models**					
miR-223-3p	0.788	0.071	5.18 × 10^−3^	0.648	0.927
miR-142-3p	0.832	0.069	1.27 × 10^−3^	0.696	0.968
miR-155-5p	0.742	0.094	0.019	0.558	0.927
miR-486-5p	0.718	0.096	0.034	0.530	0.906
miR-125a-5p	0.782	0.074	6.21 × 10^−3^	0.636	0.928
miR-146a-5p	0.785	0.083	5.67 × 10^−3^	0.622	0.948
cfDNA	0.722	0.102	0.029	0.523	0.921
mtDNA	0.832	0.069	1.09 × 10^−3^	0.698	0.966
**Multivariate model 1** (all 6 miRNAs)	0.796	0.081	4.20 × 10^−3^	0.638	0.954
**Multivariate model 2** (all 6 miRNAs, cfDNA, and mtDNA)	0.975	0.021	4.45 × 10^−6^	0.934	1.000
**Multivariate model 3** (mtDNA and miR-142-3p)	0.833	0.069	1.17 × 10^−3^	0.697	0.968
**Multivariate model 4** (mtDNA, miR-142-3p, and miR-223-3p)	0.833	0.070	1.17 × 10^−3^	0.696	0.970
**Multivariate model 5** (mtDNA, miR-142-3p, miR-223-3p, and miR-146a-5p)	0.903	0.046	8.37 × 10^−5^	0.813	0.993

* All adjusted for age and gender (male). ^a^ Under the nonparametric assumption. ^b^ Null hypothesis: true area = 0.5.

## Data Availability

The data will be available based on reasonable request.

## Supplementary Materials

The supporting information can be downloaded at: https://www.mdpi.com/article/10.3390/ijms23179947/s1.
