# Supplementary material for: Mitochondrial DNA Together with miR-142-3p in Plasma Can Predict Unfavorable Outcomes in Patients after Acute Myocardial Infarction
Source: Int J Mol Sci. 2022 Sep 1;23(17):9947. doi: 10.3390/ijms23179947 (PMC9456000; doi:10.3390/ijms23179947)
Supplement: Supplementary file 1 [file ijms-23-09947-s001.zip › ijms-1893834-supplementary.pdf]

Mitochondrial DNA Together with miR-142-3p in Plasma Can Predict Unfavorable Outcomes in Patients after Acute Myocardial Infarction

Teodora Barbălată [1], Alina I. Scărlătescu [2,3], Gabriela M. Sanda [1], Laura Toma [1], Camelia S. Stancu [1], Maria Dorobanțu [2,3], Miruna M. Micheu [3], Anca V. Sima [1,*], Loredan S. Niculescu [1,*]

1 Lipidomics Department, Institute of Cellular Biology and Pathology "Nicolae Simionescu" of the Romanian Academy, 8, B.P. Hasdeu Street, 050568 Bucharest, Romania
2 Department of Cardiology, "Carol Davila" University of Medicine and Pharmacy, 050474 Bucharest, Romania
3 Department of Cardiology, Clinical Emergency Hospital of Bucharest, 014461 Bucharest, Romania
* Correspondence to anca.sima@icbp.ro (A.V.S.); loredan.niculescu@icbp.ro (L.S.N.)

Supplementary Tables

Table S1. **Lipid metabolism-related parameters** and glucose in plasma of ST-segment elevation myocardial infarction (STEMI) patients at T0, T1 and T6 time points and healthy subjects (Control).

Parameters	Control	STEMI-T_0	STEMI-T_1	STEMI-T_6
Total cholesterol (mg/dL)	204.99 ± 7.42	216.35 ± 5.55	184.19 ± 6.85$^{###}$	140.36 ± 5.06$^{***, ###, \$\$\$}$
HDL-C (mg/dL)	43.60 ± 3.33	28.91 ± 0.97***	25.16 ± 0.81$^{***, ##}$	27.28 ± 0.84$^{***, \$}$
LDL-C (mg/dL)	152.21 ± 8.50	156.81 ± 4.84	123.21 ± 5.69$^{##}$	74.46 ± 3.51$^{***, ###, \$}$
Triglycerides (mg/dL)	78.30 ± 7.94	143.80 ± 7.06**	145.80 ± 7.79***	165.78 ± 11.76***
apoA-I (mg/dL)	117.72 ± 8.50	88.00 ± 2.98***	92.80 ± 4.06***	61.18 ± 3.02$^{***, ###, \$\$\$}$
apoE (mg/dL)	2.02 ± 0.25	2.47 ± 0.10	2.27 ± 0.09	2.00 ± 0.12$^{#}$
apoJ (μg/mL)	233.83 ± 10.32	203.71 ± 4.67**	224.54 ± 7.34$^{#}$	222.85 ± 3.79$^{##}$
Lp(a) (μg/mL)	101.94 ± 9.65	NA	114.07 ± 6.15	120.95 ± 12.52
Glucose (mg/dL)	92.80 ± 2.32	120.50 ± 4.13	111.84 ± 3.54$^{*,#}$	NA

Data are given as means ± SEM. Variations between the parameters of "STEMI-T0", "STEMI-T1", "STEMI-T6" and "Control" groups were analyzed by Independent Student T-test and considered statistically significant when the p value is below 0.05 (marked with * vs. Control; # vs. STEMI- T0; $ vs. STEMI-T1), or below 0.01 (marked with ** vs. Control; ## vs. STEMI- T0; $$ vs. STEMI-T1), or below 0.001 (marked with *** vs. Control; ### vs. STEMI- T0; $$$ vs. STEMI-T1). apoA-I, apolipoprotein A-I; apoE, apolipoprotein E; apoJ, apolipoprotein J; HDL-C, high-density lipoprotein cholesterol; LDL-C, low-density lipoprotein cholesterol; Lp(a), lipoprotein (a); SEM, standard error of the mean; NA, not available.

Table S2. Oxidative, inflammatory and cardiac parameters in plasma from ST-segment elevation myocardial infarction (STEMI) patients at T0, T1 and T6 time points and Controls.

Parameters	Control	STEMI-T_0	STEMI-T_1	STEMI-T_6
PON1 protein (ng/mL)	2.61 ± 0.18	2.64 ± 0.09	2.86 ± 0.08	2.64 ± 0.11
PON1 activity (U/L)	363.20 ± 60.40	242.98 ± 15.33**	463.33 ± 55.43###	286.09 ± 17.44#,$$
PON1 specific activity (U/ng)	131.10 ± 15.30	89.86 ± 4.92**	144.31 ± 21.68###	113.46 ± 6.25##
MPO protein (ng/mL)	29.51 ± 2.40	80.83 ± 4.99***	50.67 ± 3.91**	37.02 ± 2.03*,###,$
MPO activity (fmoles/min/mL)	108.38 ± 11.08	163.27 ± 12.16*	205.01 ± 14.99**	156.45 ± 11.54*
MPO specific activity (fmoles/ng/min)	4.10 ± 0.73	2.42 ± 0.16	4.23 ± 0.45###	4.29 ± 0.35###
Ceruloplasmin (μg/mL)	576.91 ± 49.56	653.08 ± 45.34	891.33 ± 38.00***,###	584.94 ± 33.12$$$
CRP (μg/mL)	1.91 ± 0.84	9.34 ± 0.86**	20.70 ± 1.73***, ###	2.25 ± 0.28*,###, $$$
LDH (U/L)	1757 ± 132	7033 ± 404***	4758 ± 273***, ###	1874 ± 114###, $$$

Data are given as means ± SEM. Variations between the parameters of "STEMI-T_0", "STEMI-T_1", "STEMI-T_6" and "Control" groups were analyzed by Independent Student T-test and considered statistically significant when the p value is below 0.05 (marked with * vs. Control; # vs. STEMI- T_0; $ vs. STEMI-T_1), or below 0.01 (marked with ** vs. Control; ## vs. STEMI- T_0; $$ vs. STEMI-T_1), or below 0.001 (marked with *** vs. Control; ### vs. STEMI- T_0; $$$ vs. STEMI-T_1). CRP, C-reactive protein; cTNNI3, cardiac troponin I3; LDH, lactate-dehydrogenase; NT-pro-BNP, N-terminal prohormone of brain natriuretic peptide; PON1, paraoxonase 1; MPO, myeloperoxidase; SEM, standard error of the mean.

Table S3. Plasma lipid metabolism-related parameters of ST-segment elevation myocardial infarction (STEMI) patients at T_0, T_1 and T_6 time points, grouped by the occurrence of subsequent major adverse cardiovascular events (MACE) at 6-month follow-up.

Parameter	STEMI-T_0		STEMI-T_1		STEMI-T_6	
	**No MACE**	**With MACE**	**No MACE**	**With MACE**	**No MACE**	**With MACE**
Total cholesterol (mg/dL)	214.03 ± 5.20	203.14 ± 13.56	179.86 ± 7.78***	175.61 ± 9.92	144.20 ± 6.26$^{\$\$\$}$	128.83 ± 6.92$^{\&\&\&}$
HDL-C (mg/dL)	30.49 ± 0.96	23.56 ± 2.88**	25.87 ± 1.01**	24.77 ± 1.51	28.03 ± 0.93	24.96 ± 1.81
LDL-C (mg/dL)	158.41 ± 4.97	140.97 ± 13.53	116.24 ± 6.28***	120.02 ± 9.11	76.16 ± 4.47$^{\$\$\$}$	69.49 ± 4.32$^{\&\&\&}$
Triglycerides (mg/dL)	136.02 ± 6.94	143.38 ± 14.26	137.33 ± 6.96	154.06 ± 18.74	163.90 ± 11.90	171.89 ± 32.82$^{\&}$
Glucose (mg/dL)	114.55 ± 4.10	126.57 ± 5.02	106.72 ± 3.38	118.21 ± 6.44	NA	NA
apoA-I (mg/dL)	87.82 ± 3.56	88.86 ± 6.78	94.87 ± 4.97	92.87 ± 10.02	60.49 ± 3.39$^{\$\$\$}$	63.34 ± 7.49$^{\&}$
apoE (mg/dL)	2.34 ± 0.11	2.71 ± 0.18	2.18 ± 0.10	2.29 ± 0.23	1.95 ± 0.12	2.15 ± 0.35
apoJ (μg/mL)	203.96 ± 5.12	191.52 ± 11.92	227.35 ± 7.09	230.39 ± 50.06	217.88 ± 3.75	238.18 ± 9.19$^{\%}$
Lp(a) (μg/mL)	NA	NA	105.33 ± 5.30	118.48 ± 13.62$^{\$}$	112.12 ± 14.22	134.19 ± 23.54

Data are given as means ± SEM. Variations between the parameters of "STEMI-T_0", "STEMI-T_1", "STEMI-T_6" and "Control" groups were analyzed by Independent Student T-test and considered statistically significant when the p value is below 0.05 (marked with * vs. STEMI-T_0 no MACE; # vs. STEMI-T_0 MACE; $ vs. STEMI-T_1 no MACE; & vs STEMI-T_1 MACE; % vs STEMI-T_6 no MACE). apoA-I, apolipoprotein A-I; apoE, apolipoprotein E; apoJ, apolipoprotein J; HDL-C, high-density lipoprotein cholesterol; LDL-C, low-density lipoprotein cholesterol; Lp(a), lipoprotein (a); SEM, standard error of the mean; NA, not available.

Table S4. Oxidative, inflammatory and cardiac parameters in plasma of ST segment elevation myocardial infarction (STEMI) patients at T0, T1 and T6 time points, grouped by the occurrence of subsequent major adverse cardiovascular events (MACE) at 6-month follow-up.

Parameter	STEMI-T_0		STEMI-T_1		STEMI-T_6	
	**No MACE**	**With MACE**	**No MACE**	**With MACE**	**No MACE**	**With MACE**
PON1 protein (ng/mL)	2.54 ± 0.10	2.82 ± 0.16	3.04 ± 0.09**	2.62 ± 0.12$^{\$}$	2.72 ± 0.14	2.39 ± 0.14
PON1 activity (U/L)	233.59 ± 16.24	254.03 ± 37.67	500.32 ± 60.27***	252.84 ± 46.79$^{\$}$	292.81 ± 20.73$^{\$\$\$}$	264.25 ± 31.58
MPO protein (ng/mL)	74.44 ± 5.13	103.10 ± 14.21*	45.53 ± 3.45***	65.29 ± 13.04$^{\$}$	37.92 ± 2.51	34.01 ± 2.64$^{\&}$
MPO activity (fmoles/min/mL)	152.09 ± 12.48	185.45 ± 28.06	199.36 ± 15.80*	237.62 ± 40.82	151.01 ± 12.06$^{\$\$}$	174.24 ± 174.24
Ceruloplasmin (µg/mL)	624.59 ± 57.38	638.25 ± 109.45	893.50 ± 42.25***	793.65 ± 30.25$^{\#}$	559.41 ± 27.74$^{\$\$\$}$	747.50 ± 184.10$^{\&}$
CRP (µg/mL)	8.10 ± 0.76	12.00 ± 2.30*	17.17 ± 1.61***	34.98 ± 4.63$^{\#\#\#,\$\$\$}$	1.85 ± 0.26$^{\$\$\$}$	3.67 ± 0.75$^{\&\&\&,\%\%}$
LDH (U/L)	6995 ± 455	7763 ± 958	4454 ± 259***	6746 ± 125$^{\$\$\$}$	1995 ± 127$^{\$\$\$}$	1479 ± 225$^{\&\&\&}$

Data are given as means ± SEM. Variations between the parameters of "STEMI-T_0", "STEMI-T_1", "STEMI-T_6" and "Control" groups were analyzed by Independent Student T-test and considered statistically significant when the p value is below 0.05 (marked with * vs. STEMI-T_0 no MACE; # vs. STEMI-T_0 MACE; $ vs. STEMI-T_1 no MACE; & vs STEMI-T_1 MACE; % vs STEMI-T_6 no MACE). PON1, paraoxonase 1; MPO, myeloperoxidase; SEM, standard error of the mean.

Table S5. Prediction of subsequent major adverse cardiovascular events (MACE) in ST-elevation myocardial infarction (STEMI) patients by using the binary logistic regression (BLR) analysis using parameters associated with clinical data, lipid-metabolism, oxidative and inflammatory stress measured in plasma at hospital discharge (T_1).

Parameter	Wald χ^2 value	p value	OR (95% CI)	Model χ^2 value	Model p-value
**BLR model 1 – Lipid metabolism**					
HDL-C/apoA-I ratio	3.680	0.055	2.65 x10^{-9} (1.00 x10^{-13} - 1.535)	17.52	1.53 x10^{-3}
Atherogenic coefficient	3.478	0.062	1.453 (0.981 - 2.153)		
Age	0.271	0.603	1.023 (0.939 - 1.115)		
Gender (male)	0.111	0.739	1.600 (0.100 - 25.464)		
**BLR model 2 – Oxidative stress**					
PON1	1.572	0.577	0.417 (0.019 - 9.069)	22.58	9.48 x10^{-4}
PON1 activity	0.003	0.791	0.999 (0.992 - 1.006)		
MPO	0.025	0.100	1.042 (0.992 - 1.094)		
MPO activity	0.010	0.627	0.995 (0.977 - 1.014)		
Age	0.085	0.258	0.909 (0.769 - 1.073)		
Gender (male)	4.856	0.353	90.71 (6.66 x10^{-3} - 1.23 x10^{+6})		
**BLR model 3 – Inflammatory stress**					
CRP	3.974	0.046	1.114 (1.002 - 1.240)	32.96	1.22 x10^{-6}
LDH	3.539	0.060	1.001 (1.000 - 1.002)		
Age	5.332	0.021	0.854 (0.747 - 0.976)		
Gender (male)	1.660	0.198	0.169 (0.011 - 2.527)		
**BLR model 4 – Clinical data and risk factors**					
LVEF	4.354	0.037	0.931 (0.871 - 0.996)	52.32	1.46 x10^{-8}
Dyslipidemia	1.624	0.202	3.271 (0.529 - 20.234)		
Hyperglycemia	5.343	0.021	4.733 (1.267 - 17.685)		
Hypertension	1.043	0.307	1.997 (0.529 - 7.536)		
Obesity	0.011	0.915	0.932 (0.255 - 3.404)		
Smoking	0.314	0.575	1.583 (0.317 - 7.894)		
Age	0.145	0.703	1.516 (0.178 - 12.922)		
Gender	2.148	0.143	0.967 (0.925 - 1.011)		

HDL-C, high-density lipoprotein cholesterol; apoA-I, apolipoprotein A-I; BLR, binary logistic regression; CRP, C-reactive protein; LDH, lactate-dehydrogenase; LVEF, left ventricular ejection fraction; MPO, myeloperoxidase; PON1, paraoxonase 1; OR, odd ratio.

Table S6. Prediction of subsequent major adverse cardiovascular events (MACE) in ST-elevation myocardial infarction (STEMI) patients by binary logistic regression (BLR) analysis using miRNAs, total cell-free DNA (cfDNA) and cell-free mitochondrial DNA (mtDNA) levels measured in plasma at hospital discharge (T_1).

Parameter	Wald χ^2 value	p value	OR (95% CI)	Model χ^2 value	Model p-value
**BLR model 1**				20.82	1.97 x 10^{-3}
miR-223-3p	0.308	0.579	0.281 (0.003 – 24.787)		
miR-142-3p	0.885	0.347	10.31 (0.080 – 1.33 x10^{+3})		
miR-155-5p	0.339	0.560	0.202 (9.24 x10^{-4} – 44.050)		
miR-486-5p	3.614	0.057	1.95 x10^{-2} (3.385 x10^{-4} – 1.130)		
miR-125a-5p	3.791	0.052	42.03 (0.975 – 1.81 x10^{+3})		
miR-146a-5p	2.217	0.137	94.26 (0.237 – 3.74 x10^{+4})		
**BLR model 2**				40.57	2.50 x 10^{-6}
miR-223-3p	0.490	0.484	1.92 x10^{-2} (3.02 x10^{-7} – 1.22 x10^{+3})		
miR-142-3p	0.061	0.805	3.45 (1.85 x10^{-4} – 6.40 x10^{+4})		
miR-155-5p	1.578	0.209	6.29 x10^{+3} (7.45 x10^{-3} – 5.31 x10^{+9})		
miR-486-5p	3.117	0.078	4.79 x10^{-5} (7.67 x10^{-10} - 2.99)		
miR-125a-5p	2.296	0.130	272.73 (0.193 - 3.85 x10^{+5})		
miR-146a-5p	2.828	0.093	4.60 x10^{+3} (0.248 - 8.547 x10^{+7})		
cfDNA	2.136	0.144	0.997 (0.994 - 1.001)		
mtDNA	3.864	0.049	1.055 (1.000 - 1.112)		
**BLR model 3**				41.74	8.32 x10^{-6}
miR-223-3p	0.587	0.444	7.20 x10^{-4} (6.69 x10^{-12} - 7.87 x10^{+4})		
miR-142-3p	0.175	0.675	68.10 (1.78 x10^{-7} - 2.60 x10^{+10})		
miR-155-5p	0.960	0.327	4.22 x10^{+4} (2.34 x10^{-5} - 7.60 x10^{+13})		
miR-486-5p	2.067	0.151	4.93 x10^{-6} (3.87 x10^{-13} - 84.76)		
miR-125a-5p	1.804	0.179	2.11 x10^{+3} (0.030 -1.49 x10^{+8})		
miR-146a-5p	2.634	0.105	1.56 x10^{+5} (0.083 - 2.91 x10^{+11})		
cfDNA	1.826	0.177	0.996 (0.990 - 1.002)		
mtDNA	2.595	0.107	1.081 (0.983 - 1.190)		
Age	0.893	0.345	0.899 (0.722 - 1.121)		
Gender (male)	0.093	0.761	1.992 (0.024 - 168.25)		

BLR, binary logistic regression; cfDNA, cell-free DNA; mtDNA, mitochondrial DNA; OR, odd ratio.

Table S7. Primers sequences used for measurement of cfDNA and mtDNA by real-time in plasma of ST-segment elevation myocardial infarction (STEMI) patients.

Gene	Primer sequence 5'-3'	Gene ID	Amplicon size (bp)
LINE1	Forward: 5′-TTGCGGCACTATTCACAATAGC-3′<br>Reverse: 5′-GTGCCACATTTTCTTAATCCAGTCT-3′<br>TaqMan Probe:<br>5′-FAM-TGGAACCAACCCAAATGTCCAACAATGA-TAMRA-3′	AH005269.2	83
MT-ND1	Forward: 5′-GGCTATATACAACTACGCAAAGGC-3′<br>Reverse: 5′-GGTAGATGTGGCGGGTTTTAGG-3′	NC_012920.1	117

LINE1, long interspersed element-1; MT-ND1, Mitochondrially Encoded NADH:Ubiquinone Oxidoreductase Core Subunit 1.

Supplementary Figures

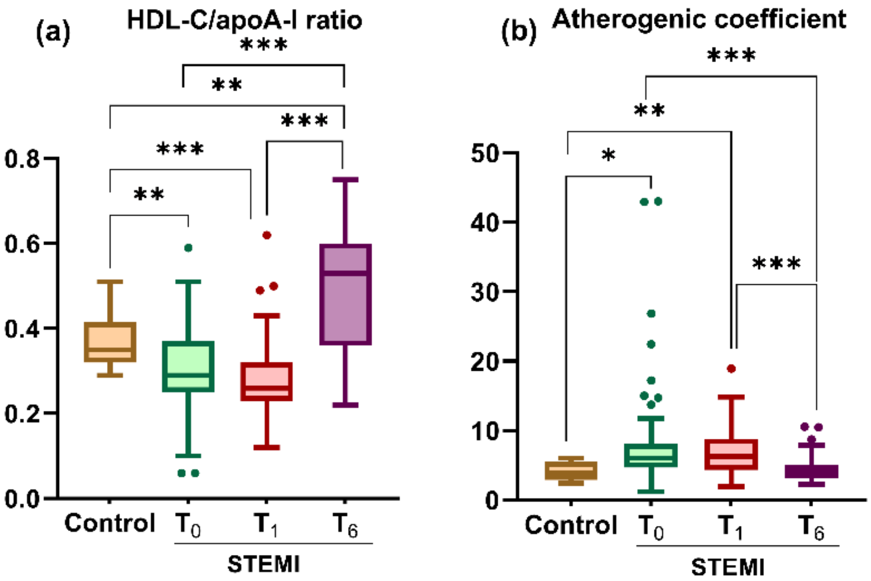

Figure S1. Ratio of plasma HDL-C/apoA-I (**a**) **and atherogenic coefficient** (**b**) for ST-segment elevation myocardial infarction (STEMI) patients at T_0, T_1 and T_6 time points and in plasma of healthy subjects (Control). Data are illustrated as boxplots with Tukey whiskers and median line. *p<0.05; **p<0.01; ***p<0.001.

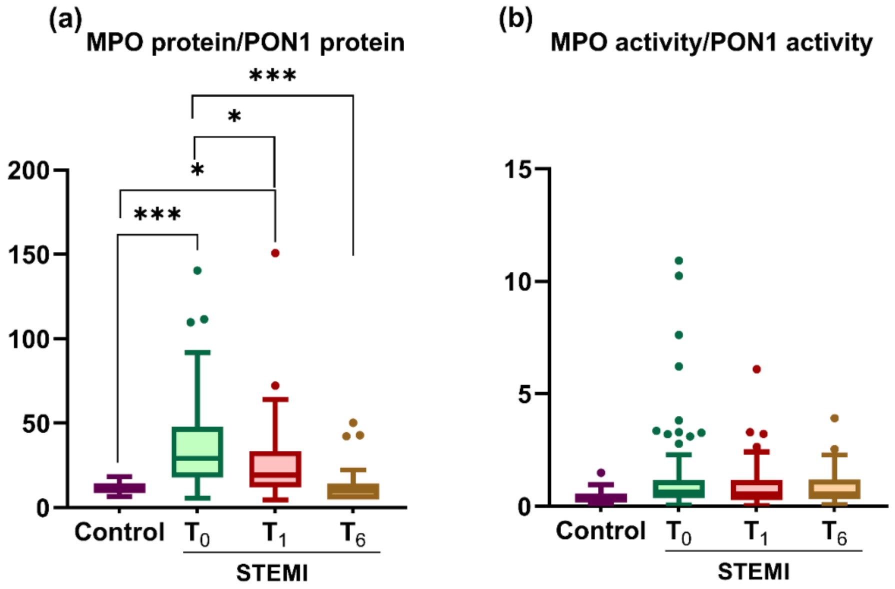

Figure S2. Levels of MPO protein/PON1 protein ratio (**a**) **and MPO activity/PON1 activity ratio** (**b**) in the plasma of ST-segment elevation myocardial infarction (STEMI) patients at T_0, T_1 and T_6 time points compared to healthy controls. Data are illustrated as boxplots with Tukey whiskers and median line. *p<0.05, **p<0.01, ***p<0.001.

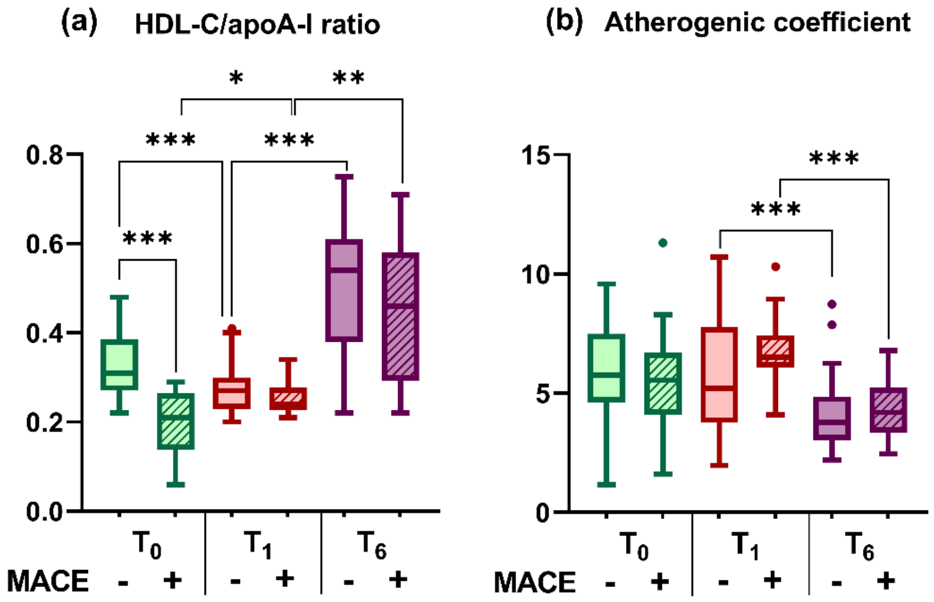

Figure S3. Variation of HDL-C/apoA-I ratio (**a**) **and the atherogenic coefficient** (**b**) in the plasma of ST-segment elevation myocardial infarction (STEMI) patients at T_0, T_1 and T_6 time points, grouped by the occurrence of subsequent major adverse cardiovascular events (MACE) at 6-month follow-up. Data are illustrated as boxplots with Tukey whiskers and median line. *p<0.05, **p<0.01, ***p<0.001.

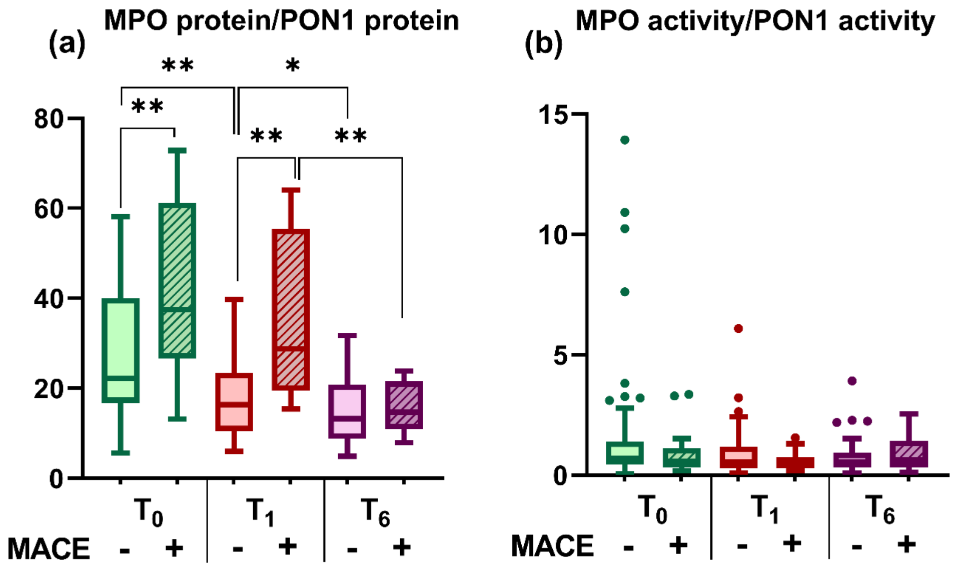

Figure S4. Levels of MPO/PON1 protein ratio (**a**) **and MPO/PON1 activity ratio** (**b**) in the plasma of ST-segment elevation myocardial infarction (STEMI) patients at T_0, T_1 and T_6 time points, grouped by the occurrence of subsequent major adverse cardiovascular events (MACE) during a 6-month follow-up. Data are illustrated as boxplots with Tukey whiskers and median line. *p<0.05, **p<0.01, ***p<0.001.
